# Using the Theoretical Domains Framework to identify strategies to support the implementation of the guidelines for the physiotherapy management of people with spinal cord injury: a qualitative study

**DOI:** 10.1038/s41394-025-00719-9

**Published:** 2025-08-28

**Authors:** KE Tranter, LA Harvey, J. Chu, J. Ilha, M. Ben, LW Chen, JV Glinsky

**Affiliations:** 1 The John Walsh Centre for Rehabilitation Research, University of Sydney, Kolling Institute, Camperdown, NSW Australia; 2 Physiotherapy Department, Universidade do Estado de Santa Catarina, Florianópolis, Brazil; 3 NSW Spinal Outreach Service, Royal Rehab, Sydney, NSW Australia; 4 Spinal Injuries Unit, Royal North Shore Hospital, Sydney, NSW Australia

**Keywords:** Rehabilitation, Translational research

## Abstract

**Study design:**

Qualitative design.

**Aim:**

The aim of the study was to (i) determine the perspectives of therapists and people with spinal cord injury (SCI) on our recently developed Australian and New Zealand Clinical Practice Guidelines for the physiotherapy management of people with SCI, and (ii) understand the barriers and facilitators to the rollout of the Guidelines and identify implementation strategies to support their uptake.

**Setting:**

Hospital and community SCI services, Australia.

**Methods:**

Twenty-one therapists and 10 people with SCI were interviewed one-on-one or within focus groups. The interviews captured perspectives of therapists and people with SCI on the Guidelines as well as barriers and facilitators to the rollout of the Guidelines. The interviews with therapists were guided by the Theoretical Domains Framework (TDF). All interviews were transcribed and thematically coded. Barriers and facilitators were then linked to the COM-B model (Capability, Opportunity, Motivation – Behaviour) to identify implementation strategies.

**Results:**

The barriers to implementation of the Guidelines identified by therapists were lack of knowledge and skill, lack of resources and challenges associated with working within large organisations. Facilitators to the uptake of the Guidelines were providing education, skill training, Guideline champions, awareness, and two-way communication between patient and therapist. Fourteen implementation strategies were identified.

**Conclusion:**

Both therapists and people with SCI thought the Guidelines were a useful tool to improve the quality of care provided to people with SCI across various health settings. Facilitators identified in this study are being used to guide current implementation projects to enhance the uptake of the Guidelines.

## Introduction

Australian and New Zealand Clinical Practice Guidelines for the physiotherapy management of people with spinal cord injury (SCI) have been recently developed (www.sciptguide.com) [1]. They contain over 100 recommendations and consensus statements that are based on rigorous reviews of the available evidence. The implementation of recommendations made in the Guidelines is important to deliver best patient care and to maximise health outcomes and functional abilities for people with SCI. However, the implementation of evidence into clinical practice is often delayed due to the complex nature of health systems. For this reason, the translation of evidence into the clinical setting requires implementation strategies that target barriers and facilitators to enable Guidelines adoption.

Implementation science improves the systematic uptake of evidence-based therapies into routine clinical practice [2]. The translation of evidence-based therapies from guidelines into the clinical setting requires behavioural change that targets the specific barriers and facilitators to practice. Tailored behaviour change interventions, that are guided by implementation theories, are effective in targeting specific determinants of practice [3–5] and have been widely used to increase knowledge translation in other health conditions [6–11]. Behaviour change interventions may target therapists, stakeholders, people with health conditions or the wider population. Behaviour change is more likely to occur if the implementation strategies target specific barriers and facilitators to practice that have been identified by a rigorous theoretical assessment. For example, audit feedback has been shown to be an effective implementation strategy to increase clinicians' adherence to recommendations made in guidelines in other areas of neurological rehabilitation [12, 13].

The Theoretical Domains Framework (TDF, v2) is a useful theory-based implementation tool which identifies influences on behaviours to better understand specific barriers and facilitators to practice [14, 15]. The TDF has been used extensively in the literature to help inform implementation strategies [10, 16] and has been found to be more effective than simple guideline dissemination alone [17]. In the SCI literature, the TDF has been used to develop self-management programs, change bowel care practices and codevelop physiotherapist-led physical activity interventions [18–20]. The TDF contains 14 different domains that can be used to guide interviews, focus groups and / or surveys to highlight key barriers and facilitators to implementation. These are then mapped against behaviour change interventions, using a framework such as the COM-B model, to identify specific implementation strategies that can be used in an attempt to increase the uptake of the Guidelines [3, 21].

Widespread uptake of recommendations from clinical guidelines is generally poor across all health conditions [22], and there is no guarantee that the recommendations from The Australian and New Zealand Clinical Practice Guidelines for the physiotherapy management of people with SCI will be implemented in practice. Therefore, the aim of this study was to (i) determine the perspectives of therapists’ and people with SCI on our recently developed Guidelines for the physiotherapy management of people with SCI, and (ii) understand the barriers and facilitators to the uptake of the Guidelines and identify implementation strategies to support the rollout.

## Method

### Design

Semi-structured focus groups and interviews were conducted for therapists (therapist-participants) and people with SCI (consumer-participants), respectively. Potential participants were contacted by phone or email and invited to participate. A sample of convenience was used for both therapist- and consumer-participants. Therapists came from varied settings such as public and private spinal services, Universities, consumer organisations and private/community services. Consumers were recruited from acute, rehabilitation and community settings. All participants provided written consent.

All participants were provided with an electronic or paper copy of the Guidelines. They were taken through the Guideline process in brief and the different types of recommendations were explained to them. They were given the opportunity to discuss the Guidelines and encouraged to ask questions prior to the focus groups or interviews.

Ethical approval was obtained from the Northern Sydney Local Health District Human Research Ethics Committee (HREC approval number 2022/ETH01217). All participants provided informed consent prior to participating in the study. The study was conducted in accordance with the relevant guidelines and regulations.

### Participants

#### Consumer-participants

Consumers were eligible to participate if they had sustained a SCI and had received physiotherapy services. There were no limitations to the severity of SCI or time since injury. Consumers were excluded if they were unable to cooperate or did not have sufficient English.

#### Therapist-participants

Therapists were eligible to participate if they provided, physiotherapy or exercise physiology SCI services (frequently or infrequently), education for the physiotherapy management of people with SCI, or were involved in the governance of physiotherapy services for the management of people with SCI across all states of Australia. Therapists were excluded if they did not have sufficient English or were involved in the development of the Guidelines.

### Interviews and focus groups

An independent professional agency (ARTD), with extensive experience in qualitative health research, was engaged to conduct the focus groups, interviews and to code the data, minimising bias. Online focus groups and interviews were conducted via Microsoft teams (Classic version).

#### Interviews with consumer-participants

Consumers were interviewed on a one-on-one basis online or over the phone. The ARTD representative used an interview guide developed by the research team. It included 13 questions to explore their perspectives on the Guidelines and how to increase uptake in the SCI community (see Supplementary File 1).

#### Focus groups with therapist-participants

Therapists participated in focus groups that were conducted online. The ARTD representative used an interview guide developed by the research team. The interview guide was predetermined using the TDF to capture therapists’ perspectives of the recommendations and the potential barriers and facilitators to implementation. It included 20 key questions across seven domains from the TDF (see Supplementary File 2). Questioning within the focus groups ensured all participants had an opportunity to respond openly. The ARTD representative was highly experienced and cognisant of the need to ensure everyone in the group had an equal opportunity to speak. Any differences in opinion were welcomed and managed in a respectful manner.

### Data management and analysis

Data were managed and analysed using NVivo software [23]. All interviews and focus groups were recorded, transcribed verbatim and then analysed according to stakeholder type. A coding framework was developed based on the TDF to extrapolate barriers and facilitators from the interviews. The TDF coding framework was developed by KET, JVG, LAH, and two ARTD representatives. The TDF framework had three tiers. The first captured the TDF domains and the second captured sub-categories of these domains. The third-tier categorised themes as either positive, negative or both. Three people coded the transcripts, two representatives from ARTD and one of the authors (KET). One transcript was independently coded by all three coders to detect similarities and differences in coding. Any differences in opinion were resolved with group discussion between the coders and applied to the other transcripts. Following this, all three coders independently coded the remaining interviews and came together to discuss any uncertainties. Once all the transcripts had been coded the data were then thematically analysed according to the TDF to identify influences on clinical practice. Illustrative quotes were extracted where appropriate. The analysis was then reviewed by all three coders and two other authors (JVG and LAH). The analysed data allowed the TDF codes to be mapped to the COM-B model to provide a list of key suggested strategies to address the specific determinants of practice identified.

## Results

### Flow of participants through the study

Ten consumers participated in one-on-one interviews. Interview duration ranged from 20 min up to 45 min. Consumers were primarily 5 years or less post injury, or greater than 15 years. Twenty-one therapists (20 physiotherapists and one exercise physiologist) participated in one of four online focus groups. Focus groups consisted of between three to seven therapists and ran between 55 to 90 min. More than half of therapists had 6 or more years’ experience working with people with SCI. The characteristics of participants can be viewed in Table 1.

**Table 1:** Characteristics of therapist-participants and consumer-participants.

	Therapist-participants (*n* = 21)	Consumer-participants (*n* = 10)
Gender (M:F)	3:18	8:2
Time qualified as therapist (years): n(%)		
<1 year	0 (0)	
1–5	2 (10)	
6–10	8 (38)	
11–15	3 (14)	
>15	8 (38)	
Experience working with people with SCI (years): n(%)		
<1 year	1 (5)	
1–5	8 (38)	
6–10	6 (29)	
11–15	3 (14)	
>15	3 (14)	
Primary workplace (n) (Inpatient: Community: University)	10:9:2	
ASIA^a^ impairment scale n(%)		
C1-4 AIS A,B,C,D		4 (40)
C5-C8 AIS A,B,C,D		4 (40)
T1-S5 AIS A,B,C,D		2 (20)
Time post injury (years)		
<1 year		4 (40)
1–5		3 (30)
6–10		0 (0)
11–15		0 (0)
>15		3 (30)

^a^American Spinal Injuries Association (as per the International Standards for Neurological Classification of Spinal Cord Injury, ISNCSCI).

### Main findings

Key perspectives were identified in the consumer data. Representative quotations or discussion points that captured their perspectives are summarised in Table 2. Key themes were identified in the therapist data. These themes were summarised according to domains of the TDF. They were then matched against behaviour change interventions to identify implementation strategies that could be used to facilitate the uptake of the Guidelines. These are summarised in Table 3.

**Table 2:** Summary of key perspectives of consumer-participants.

Theme / Perspective	Quotation / Discussion point
Useful resource for therapists	*It gives guidelines … to some that are not as experienced. And it’s great that you give them this framework to work with, rather than trying to dig everything up or ring their mentor every five minutes to find something out. – Consumer-participant (C08)*
Importance of having the Guidelines as an educational resource to build therapists’ knowledge and awareness.	*As far as I’m concerned, it is probably one of the greatest things I’ve seen happen. Because if you are situated at, shall we say Broken Hill, how many spinal cord issues would you see on a regular basis where they couldn’t be moved to a bigger hospital? And I’m just using Broken Hill as just a place, but it’s remote enough. – Consumer-participant (C03)*
Consistency among therapists	*I think it’s a fantastic idea because what various therapists do varies from one physiotherapist to the next physiotherapist. Which I find quite interesting, actually. I think Guidelines would be a very, very useful tool. – Consumer-participant (C03)*
Consumers will be better informed of physiotherapy interventions.	*If people are interested, they can see what’s recommended for treatment. Everything that they either know or they don’t know that might be good for them or helpful for them, they can have a look at what’s there. – Consumer-participant (C01)*
Education about the Guidelines for people with SCI	*…once a week (in hospital) there’ll be a get together and it will be like an in-house info thing for the patients, and they’ll put somebody up there. So it could be brought up as something like that. – Consumer participant (C08)*
Increasing awareness through promotion	Consumers suggested circulating the Guidelines through online information sessions, SCI organisations, newsletters or social media platforms.
Health Literacy	Consumers felt that the various levels of literacy and health literacy would impact someone’s ability to interpret the Guidelines.
Readiness for people with SCI to engage	”*It’s pretty hard. Patients are often distressed, and they don’t want to always sit down and read through, lots of text and conditions and that sort of thing… but basically whatever you do needs to reinforce the need for patient confidence in the therapist and the treatment.” – Consumer participant (C07)*
Therapists to tailor the recommendations of the Guidelines to the needs of each individual patient in collaboration with the patient	*It’s a starting point anyway. Cause every single one of your clients is going to be different. – Consumer-participant (C06)*
	*You need to remember to treat the patient based upon what their needs are as well and match the needs. Use the Guidelines based on the patients’ needs rather than try to get patients to fit the Guidelines themselves. – Consumer-participant (C01)*

**Table 3:** Facilitators (+) and Barriers (−) to the implementation of the Guidelines according to domains of the Theoretical Domains Framework (TDF) and recommended Implementation strategies as per the COM-B model.

TDF^a^ Domain	Theme	Facilitator (+)/Barrier (−)	Implementation Strategy (COM-B Model)
Knowledge	Awareness of the Guidelines	+/−	C: Education
	Increasing fidelity of treatment by summarising evidence	+	C: Education
	Lack of knowledge to carry out certain interventions	−	C: Education; training
	Knowledge dissemination through education	+	C: Education
	Health literacy of consumers	+/−	C: Education
	Lack of understanding of the hierarchy of recommendations	−	C: Education
	Limited baseline knowledge and ability to clinically reason	−	C: Education
Skill	Supportive training	+	C: Training
	Lack of training and experience with interventions	−	C: Training; feedback and monitoring
Beliefs about capabilities	Therapists’ confidence with intervention delivery	+/−	C: Training
Intention	The Guidelines are perceived as useful for clinical practice	+	M: Incentivisation; persuasion
	Intent to follow recommendations in context of own clinical reasoning	+/−	C: Education; M: Persuasion
Environmental context and resources	Organisational support to facilitate networking and collaboration	+	O: Enablement; social support
	Reflective clinical practices	+	O: Enablement; social support
	Supportive workplace and team structures	+	O: Enablement; social support
	Internal processes	+/−	O: Social support
	Access to resources	+/−	O: Enablement
	Lack of dedicated time for education and training to upskill	−	O: Social support; enablement
	Funding, time, access to equipment/SCI services will impact a therapists’ ability to adhere to the Guidelines	+/−	O: Social Support; enablement
Social / professional role and identity	Evidence based justification for practices	+	C: Education; M: Persuasion
	Reach of Guidelines needs to include other disciplines who might use the Guidelines (other than physiotherapists)	+	C: Education; M: Persuasion
Beliefs about consequences	Education and collaboration with person with SCI	+	C: Education O: Enablement; M: Modelling
	Reinforce current practice	+	O: Enablement; M: Modelling
	Behaviour change difficult in context of entrenched beliefs and systems	−	M: Coercion; C: Education
Memory, attention and decision processes	Guidelines engrained in the handover process	+	O: Enablement; environmental restructuring
	Assist prioritisation of service provision and resource utilisation	+	O: Enablement; environmental restructuring
Emotion	Positivity of usefulness of Guidelines to aid in the delivery of best practice	+	O: Enablement; M: Persuasion; modelling

^a^TDF Theoretical Domains Framework.

*COM-B* COM-B behaviour change model, *C* capability, *O* opportunity, *M* motivation, *B* Behaviour.

#### Theme 1: Knowledge and awareness of the Guidelines

##### Subtheme 1.1: Therapists’ knowledge [TDF construct: Knowledge]

The therapists’ identified the need for education to build their knowledge as the most important strategy required to promote the widespread uptake of the Guidelines. It was also recognised that education needs to begin with university students or with new or inexperienced staff that are supported by senior clinicians that lead by example.

*Particularly with the incomplete spinals from non-traumatic causes, they pop up all over the place…So I think the challenge will be making sure we cast the information as broadly as we can so that the people that need it have access to it and they feel supported. – Physiotherapist P03 (Focus group 1)*

Knowledge of the Guidelines and the underpinning evidence was deemed important. It was noted that these Guidelines may make different recommendations than Guidelines for other clinical areas. This could be challenging for therapists that work across clinical specialities.

*An example would be our stroke Guidelines don’t recommend the routine use of stretch (for the)…prevention of contracture, where in these Guidelines that was an…(evidence-based recommendation FOR the use of stretch) – Physiotherapist P05 (Focus group 3)*

Respiratory interventions were frequently discussed as a sub-specialty requiring further guidance and education. Several therapists said they would require further training to implement the respiratory related recommendations or to be able to provide respiratory management plans.

*We’re always desperate from an acute care perspective to get Guidelines particularly around respiratory management - Physiotherapist P02 (Focus group 2)*

*We’ve just started to implement a bit more inspiratory muscle training… I think us, as clinicians, need education on how to progress that particular intervention for it to be effective. And we need to understand more about the evidence for that particular intervention. – Physiotherapist P02 (Focus group 2)*

##### Subtheme 1.2: Therapists’ willingness to follow the guidelines [TDF constructs: Intentions, Beliefs about capabilities]

Generally, therapists expressed a willingness to follow the recommendations made by the Guidelines, provided they could exercise their own clinical reasoning that considered the individual needs of their patients. Some therapists did express that they weren’t in full agreeance with some recommendations. The clinicians acknowledged that in general the recommendations were appropriate, however they were concerned that they may deter therapists from considering certain treatments, and that these interventions could be provided in specific clinical scenarios.

*I just really worry a little bit that if we… make a really big statement… it could get misused by people who don’t have the expertise to see the refinement there. That in some situations it may be indicated to actually range a person, stretch a person, even just check that they can still have their arms moved with a daily physio assessment when they’re doing their chest or something like that. – Physiotherapist P05 (Focus group 3)*

They reported that the Guidelines would be a useful resource to assist in justifying interventions to patients, families and carers as well as funding bodies.

*We’re providing them with the best information, or with thorough and detailed information to inform their choices around how they spend their money, and where they put their energy and spend their therapy time – Physiotherapist P03 (Focus group 1)*

#### Theme 2: Intent for implementation of the Guidelines

##### Subtheme 2.1: Therapists’ intent to implement [TDF constructs: Knowledge, Skills, Intent, Beliefs about capabilities]

The majority of therapists expressed intent to implement the Guidelines to ensure delivery of evidence-based practice, and to optimise patient outcomes and quality of life. They also acknowledged the role of the Guidelines in promoting consistency and continuity of care between therapists and services.

*…no two physios are going to do exactly the same thing, but having a consistency of treatment approach and ideas and what you’re working towards is really important… it’s that sense of fairness and Guidelines try to bring that fairness a little bit closer. – Physiotherapist P05 (Focus group 3)*

Most therapists said that it will be very important to implement the Guidelines in the workplace, as it will help therapists deliver evidence-based practice, increase the likelihood of meeting patients’ needs and improving their quality of life.

*It helps with goal setting, getting best outcomes for our patients and managing them over the continuum of care – Physiotherapist P01 (Focus group 2)*

Some therapists expressed a lack of confidence to implement interventions due to a lack of skill. Common areas identified were: respiratory management; virtual reality training and robotics; electrical stimulation; management of upper limb reconstructive surgery.

*… I wouldn’t be as confident with…virtual reality training and the robotics training, and that’s simply because of access and experience with equipment. – Physiotherapist P04 (Focus group 1)*

##### Subtheme 2.2: Skills [TDF construct: Knowledge, Skills, Beliefs about capabilities, Environmental context and resources, Social influences, Professional role and identity]

The complexity of physiotherapy care was commonly discussed. Therapists emphasised the importance of appropriate supervision, training and skills for inexperienced therapists managing people with SCI to be able to deliver best patient care.

*We do higher-grade to lower-grade supervision. You get ideas for treatments and interventions from people who’ve had experience with similar patients. Particularly as they get more complex, it’s about adapting interventions to the patients. – Physiotherapist P02 (Focus group 1)*

The importance of providing appropriate training and support was emphasised such as, practical training from more experienced therapists to understand how to implement certain interventions.

*…a new therapist may not have had exposure to (an intervention). So they may not have even used a body weight support system or the FES bike. So just ensuring that the training necessary to implement (these interventions) is done prior. – Physiotherapist P03 (Focus group 4)*

A number of therapists felt they had appropriate skills to implement the Guidelines. However, several therapists said they would require further training to implement the respiratory related recommendations or to be able to provide respiratory management plans. Informal training and support were also deemed important for upskilling inexperienced therapists. Therapists emphasised the need for networking and support for regional or rural therapists, or therapists working outside a designated SCI unit.

*…the different respiratory recommendations…having a plan for this when they do leave the rehab hospital, I think that’s something where myself, I would definitely still be looking for a lot of support from team leaders around making sure that the plan was really good and achieving the best outcome for that person. – Physiotherapist P01 (Focus group 4)*

##### Subtheme 2.3 Workplace environment and support [TDF constructs: Knowledge; Skills; Environmental context and resources; Memory, attention and decision processes; Social influences; Beliefs about consequences]

It was emphasised that for therapists to be able to implement the Guidelines they require dedicated time for education and training to upskill. A common report was how challenging it was to find this time in busy workplaces.

*Just having the actual skills to implement them appropriately and having the time, to block out time for therapists to have the training. – Physiotherapist P03 (Focus group 4)*

The therapists felt that to increase the uptake of the Guidelines it would be important to have a supportive workplace that enabled staff to access continuing professional development. They also talked about the importance of having the opportunities to collaborate with experienced colleagues (from the same and or different organisations). A common focus for therapists was the benefits of working in a flexible workplace that facilitates discussions and reflective practice to continually improve care delivery and the implementation of the Guidelines. Suggested strategies included a workplace champion to promote and advocate the Guidelines within their department, online access to Guidelines, use of Guidelines within internal training resources such as orientation manuals and policies, and patient educational programs. More broadly, other supports were suggested such as state-wide webinars and presentations, integration of Guidelines onto www.elearnSCI.org (free only training modules for healthcare professionals developed by ISCOS) [24] as well as mentoring for therapists in regional areas.

##### Subtheme 2.4 Multidisciplinary team knowledge of the guidelines [TDF construct: Social/professional role and identity]

Participants discussed the importance of the Guidelines reaching other disciplines who might also be involved in their implementation. For example, exercise physiologists may be involved in implementing strength and conditioning programs for people with SCI so need to be across relevant recommendations; or Doctors, particularly those involved in spasticity and respiratory management.

*It will be another way to educate and explain what we are doing and why, or why we are not doing something perhaps as well. – Physiotherapist P01 (Focus group 1)*

#### Theme 3: Acceptance of the Guidelines

##### Subtheme 3.1: Acceptance by people with SCI [TDF constructs: Beliefs about consequences; Environmental context and resources; Knowledge]

Consumers felt that a persons level of health literacy could impact their interpretation of the Guidelines. They also highlighted the importance of two-way communication between therapist and consumers about the recommendations of the Guidelines, so consumers can make informed decisions around their care.

*If somebody told me not to do something, I would question why they thought that as a patient, but I would accept what they told me. Because they know a lot more than I. – Consumer-participant (C03)*

Therapists reiterated this, saying that many people with SCI seek out evidence-based treatments so are therefore more likely to accept the recommendations made in the Guidelines.

*I think it will just allow me to work with the client to discuss the Guidelines and have them, as a team, as a clinician-patient team, guide our therapy…. They might be a little bit more open if they have an understanding of various interventions for one goal. – Physiotherapist P03 (Focus group 4)*

##### Subtheme 3.2: Changes to practice for therapists [TDF construct: Intentions; Social influences]

Several therapists reported that the Guidelines will continue to reinforce their current practice and provide a justification for their clinical decision-making.

*I think they’ll provide more confidence in what I already am doing. So I think you can provide your interventions with more confidence. – Physiotherapist P02 (Focus group 1)*

However, some therapists reported that the Guidelines would cause them to change their priorities for practice to ensure they are focusing on interventions with a stronger evidence base.

*Actually going into the evidence that’s been referenced and making sure that…the way that we are carrying those recommendations out is in line with the most up-to-date evidence. – Physiotherapist P04 (Focus group 1)*

Therapists mentioned the usefulness of the Guidelines to help services prioritise resources and training. One therapist said the Guidelines can be used to help them ‘justify their business case’ for funding to support the purchase of equipment.

*It will impact justify or explain our treatment plans both to the patient, their family, but also the funding body. – Physiotherapist P01 (Focus group 2)*

#### Theme 4: Promotion and distribution of the Guidelines

##### Subtheme 4.1: Promotion to therapists [TDF construct: Memory, attention, and decision processes]

Therapists and consumers agreed that education on how to interpret the Guidelines was important during promotion and distribution of the Guidelines. Therapists suggested education and internal processes to ensure dissemination of the Guidelines. While consumers suggested therapists attend information sessions run by those involved in the development of the Guidelines. In addition, consumers thought it was important to have processes in place to notify therapists of any further updates to the Guidelines. One consumer advocated that the Guidelines become part of the handover process to promote awareness among different therapists and services.

*I would say it’ll be also a good practice that each time there’s a handover to a different clinician, at different stages, medical hospital, rehab, and then in community staff, that the clinician goes over it. I don’t mean in detail, but they’ll say, “This thing’s really useful.” – Consumer-participant (C08)*

##### Subtheme 4.2: Promotion to people with SCI

Consumers reported that they and their families could become more aware of the Guidelines if they were promoted through online information sessions, SCI organisations, newsletters or social media platforms. Consumers advocated for structured inpatient information sessions.

*…at the hospital I was at, we had a lecture every week about spinal cord injuries and that would certainly be a very good topic to bring up at such a session. Most of the spinal cord patients would attend those sessions. And that would be an interesting and very good way to get the message across. – Consumer-participant (C02)*

## Discussion

Our study captured the perspectives of both therapists and consumers on the recommendations provided by the Australian and New Zealand Clinical Practice Guidelines for the physiotherapy management of people with SCI and identified barriers and facilitators to their ongoing and future rollout. Understanding these perspectives and potential determinants of practice is important as they will help guide implementation strategies designed to increase the uptake of the Guidelines. Specifically, the use of the TDF allows the mapping of targeted implementation strategies to promote uptake of the Guidelines into clinical practice.

A key determinant of practice was therapists’ knowledge of the Guidelines. Focus groups identified that senior therapists from public services had a better awareness of the Guidelines than those from non-public services. This was likely because their peers were involved in the development of the Guidelines. It was identified that dissemination of the Guidelines should target university students, junior staff, private service providers, as well as therapists in regional or rural services and adjacent areas of the multidisciplinary team. In addition, the need for structured educational sessions to support clinical decision making was highlighted. The role of specific educational sessions has been shown to improve adherence to desired practice which then positively impacts patient outcomes [25]. The way in which education is delivered will impact translation of evidence into clinical practice. Potential evidence-based strategies include the use of educational meetings, courses, seminars and workshops [25], local opinion leaders to educate other staff [26] and educational outreach visits [27] to improve the practice of therapists.

Therapists and consumers acknowledged that the rollout of the recommendations within the Guidelines required decision making by the therapists in consultation with the patient. Both groups also discussed the importance of two-way communication between patient and therapist to assist in better informed cohesive health care decisions. Ensuring patient knowledge of their health conditions and management are key to successful communications and care decisions that are aligned with best practice [28]. Whilst consumers did not express any concerns about their therapists’ choices of treatments being restricted by the Guideline, it is possible that this may play out differently in reality. If consumers are exposed to a lot of social media attention about particular treatments, they may be keen to receive these treatments irrespective of the recommendations of the Guidelines.

The implementation of recommendations of the Guidelines requires therapists to have the skills and self-belief about their capabilities to carry out an intervention. Therapists discussed interventions that they did not feel confident to administer. They felt this was due to a lack of: (i) time to upskill, (ii) resources within their workplace, and (iii) exposure to certain clinical cases. This issue is not unique to the SCI field and has been documented in other health conditions [29]. Therapists discussed the importance of networking and accessing specialised clinicians to support other therapists in such situations. Within specialised units, change in internal processes such as the use of competencies as well as the use of champions for training and supervision sessions to support skill acquisition for less experienced staff was discussed. Workplaces wanting to implement the recommendations of the Guidelines should prioritise dedicated clinical time to allow therapists to upskill through education and skills training sessions, as well as through opportunities to collaborate with colleagues. These types of strategies would improve adherence to the Guidelines over time and therefore should be part of any implementation plan.

A strength of this study was the use of the TDF to help guide the therapists’ interview scripts and to analyse the data. Understanding how the themes and subthemes relate to the TDF allows for a better understanding of the determinants of practice and is crucial for planning implementation strategies for the effective uptake of the Guidelines. This study effectively explored therapists’ and consumers’ perspectives on implementation and covered various constructs of the TDF including knowledge, intent, skills, beliefs about capabilities, beliefs about consequences, emotion, social and professional role and identity, social influence and environmental context and resources. The multiple constructs identified here shows the complex nature of implementing evidence-based practice. Implementation of an intervention is enabled by addressing the barriers and facilitators to practice identified by the TDF which are then mapped to behaviour change strategies. The use of the TDF together with behaviour change models such as the COM-B model has been widely used in the literature [30] and has been shown to be effective in creating positive change to improve adherence to desired behaviours.

One limitation of this study is that the interview questions were generalised to the whole of the Guidelines and not a specific intervention. As such the barriers and facilitators described are not necessarily related to the application of all interventions described in the Guidelines. Also, participants were only recruited from Australia. Therefore, the determinants of practice identified in this study may not be completely representative of the issues within other healthcare models more broadly. Nonetheless, the determinants of practice and the identified implementation strategies reflect important factors that can be considered for use in the environmental context to which the intervention is to be implemented. Another limitation was the use of an external agency to conduct the interviews, who are unlikely to have the content knowledge of a physiotherapist. As such, the questioning may not have been nuanced to perhaps uncover issues relating to the provision of evidence-based practice. However, an external agency provides more objective questioning and may allow more candid responses from the people being interviewed. The ARTD representative was very skilled in leading the discussions with open ended questions to tease out the issues despite them not having physiotherapy knowledge for the management of people with SCI.

Overall, this study captured varying perspectives of different therapists from various workplace settings and consumers. Both the therapist- and consumer-participants agreed the Guidelines are a useful resource and their implementation will be important for improving the consistency of care across different health services. Specifically, this study identifies key implementation strategies such as: education and skills training for therapists; access to the guidelines and specialised advice; a clinical champion to model desired practices; organisational support to promote the adoption of the guidelines within workplace processes as well as education and empowerment for people with SCI. The implementation strategies identified in this study can be adapted to the relevant clinical setting to increase the uptake of the Guidelines and will be used to guide future implementation projects.

## Supplementary information

Supplementary File 1

Supplementary File 2

## Data Availability

The datasets generated and/or analysed during the current study are available from the corresponding author on reasonable request.
