# Supplementary material for: Using the Theoretical Domains Framework to identify strategies to support the implementation of the guidelines for the physiotherapy management of people with spinal cord injury: a qualitative study
Source: Spinal Cord Ser Cases. 2025 Aug 28;11:25. doi: 10.1038/s41394-025-00719-9 (PMC12394610; doi:10.1038/s41394-025-00719-9)
Supplement: Supplementary file 1 [file 41394_2025_719_MOESM1_ESM.docx]

Supplementary File 1

1. Can you start by telling me a little about yourself and the nature of your injury?
2. How do you feel about having guidelines for physiotherapy treatment following spinal cord injury? E.g., do you think this is a good idea, or not?
3. What do you think are the positive aspects of having these Clinical Practice Guidelines?
4. What do you think are the negative aspects of having these Clinical Practice Guidelines?
5. Do you have any ideas about how we could let people with SCI know about the Guidelines and the recommendations within the Guidelines?
6. Do you feel that there is anything that we could put in place to help physios or consumers use the Clinical Practice Guidelines? For example, do you think that information sessions would be useful?
7. Are there any recommendations within the Guidelines that you strongly agree with? Explore – why?
8. Are there any recommendations within the Guidelines that you strongly disagree with? Explore – why?
9. How do you think a physiotherapist should deal with a situation where a person with SCI wants a treatment that is not recommended in the CPG?
10. How would you feel if these Guidelines recommended a treatment that you didn’t think was worthwhile?
11. How would you feel if these Guidelines did not recommend a treatment that you thought was worthwhile?
12. One recommendation is that people with SCI should not receive passive movements to improve joint mobility. What do you think about this?
13. Do you have any other thoughts about the Clinical Practice Guidelines?
