# Supplementary material for: Using the Theoretical Domains Framework to identify strategies to support the implementation of the guidelines for the physiotherapy management of people with spinal cord injury: a qualitative study
Source: Spinal Cord Ser Cases. 2025 Aug 28;11:25. doi: 10.1038/s41394-025-00719-9 (PMC12394610; doi:10.1038/s41394-025-00719-9)
Supplement: Supplementary file 2 [file 41394_2025_719_MOESM2_ESM.docx]

Supplementary File 2

1. Can you start by telling me a little about yourself and the work you do? How long have you been involved in treating people with spinal cord injuries?
2. First, we want to ask some questions so we can get an understanding about your knowledge and awareness of the Clinical Practice Guidelines.
   - 1. Were you involved in the Guidelines development process or are you new to the Guidelines?
     2. Are you aware of the recommendations relevant to your practice?
     3. Do you think your colleagues are aware that these Guidelines have been developed?
3. Let’s now explore clinical practice decisions and intent for change
   - 1. Will the Guidelines change your clinical practice? If so, how? If not, can you please tell us why, for example, you already adhere to them or you disagree with them?
     2. If you disagree with any of the recommendations, please say which one/s and why. What would it take to change your opinion?
     3. How do you seek the opinions of your colleagues about which treatments to prescribe?
     4. Do you feel people with SCI whom you treat will agree and accept the recommendations? If yes, why do you think this is? If no, what could be done to address this?
4. We’d now like to know how capable you feel in providing the recommendations, and better understand the clinical skills that you require to implement the Guidelines.
   - 1. What recommendations are you confident implementing? Explore reasons
     2. What recommendations are you not confident implementing? Explore reasons
     3. Please describe any specific skills training you may require in order to implement recommendations in the Guidelines.
5. Now we’d like to learn more about your workplace, and your role within it.
   - 1. In your workplace, who else other than your physiotherapy colleagues, may be involved with implementing the recommendations from within the Guidelines?
     2. In what ways can your workplace, including the leadership team and colleagues *[e.g., physio team, multidisciplinary team]*, support you in implementing the Guidelines?
     3. How do you feel about implementing the Guidelines for physiotherapy treatment following spinal cord injury?
6. Let’s now explore workplace adherence to the Clinical Practice Guidelines.
   - 1. How confident are you that the Clinical Practice Guidelines will be adopted in your workplace? Please describe this for us, e.g., What are the **main barriers** to adoption (e.g., resources, time, patient expectations)? Please describe any clinical priorities that would make adhering to the Guidelines difficult.
     2. What are the **main enablers** of adoption? How can adherence to the Guidelines be encouraged in your workplace? Can you think of any strategies?
     3. How important is it to you that your workplace adheres to the Clinical Practice Guidelines? Explore
     4. How will new staff in your department be made aware of the Clinical Practice Guidelines?
7. Final questions
   - 1. Is there anything else that you’d like to add that hasn’t been covered?
